# Supplementary material for: CRISPR-Cas9-based mutagenesis frequently provokes on-target mRNA misregulation
Source: Nat Commun. 2019 Sep 6;10:4056. doi: 10.1038/s41467-019-12028-5 (PMC6731291; doi:10.1038/s41467-019-12028-5)
Supplement: Supplementary file 1 — Supplementary Information [file 41467_2019_12028_MOESM1_ESM.pdf]

CRISPR/Cas9-based mutagenesis frequently provokes on-target mRNA misregulation

Rubina Tuladhar, Yunku Yeu, John Tyler Piazza, Zhen Tan, Jean Rene Clemenceau,
Xiaofeng Wu, Quinn Barrett, Jeremiah Herbert, David H. Mathews, James Kim,
Tae Hyun Hwang, and Lawrence Lum.

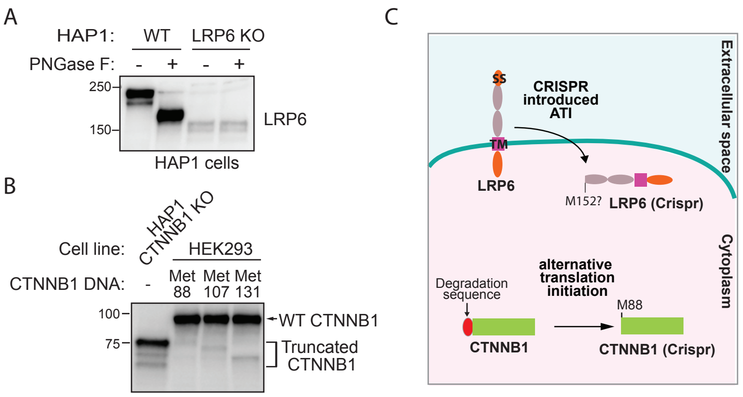

Supplemental figure 1. Inadvertent production of novel proteins by CRISPR/Cas9. (A) The LRP6 protein generated by ATI is no longer glycosylated. Lysates generated by HAP1 WT cells or with CRISPR/Cas9 editing in the LRP6 gene were incubated with deglycosidase PNGase F and subjected to Western blot analysis. **(B)** CRISPR/Cas9-edited cells express a CTNNB1 protein lacking the N-terminal GSK3β phosphorylation sites (Ser33 and Ser37) that promote β-TrCP and CTNNB1 protein turnover1. CTNNB1 cDNAs initiating at methionine 88, 107 or 131 were transiently transfected in HEK293 cells. CTNNB1 protein that initiates at methionine 88 co-migrated with the short CTNNB1 protein found in CRISPR-edited HAP1 cells. **(C)** CRISPR/Cas9 induced mislocalization of a type I transmembrane receptor. ATI results in bypassing of the signal sequence found in LRP6 WNT receptor, likely resulting in an intracellularly localized protein and produces CTNNB1 lacking degradation sequence. Source data are provided as a Source Data file.

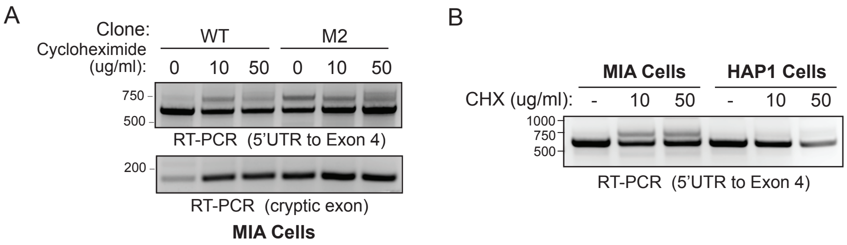

Supplemental figure 2. CRISPR/Cas9-introduced INDELs facilitate the conversion of a pseudo-mRNA to a protein encoding mRNA. (A) LKB1 pseudo-mRNA undergoes non-sense mediated decay (NMD) in MIA WT cells. MIA WT and clone M2 were incubated with 10 μg/ml or 50 μg /ml of NMD inhibitor cycloheximide (CHX). RT-PCR analysis using primers encompassing 5'UTR and exon 4 or the cryptic exon was performed. **(B)** Genomic sequence alteration by CRISPR/Cas9 gene editing must be superimposed on all the transcript variants native to different cell lines. RT-PCR using primers flanking 5' UTR and Exon 4 of LKB1 revealed an mRNA species that is sensitive to CHX in MIA cells, but not in HAP1 cells. Source data are provided as a Source Data file.

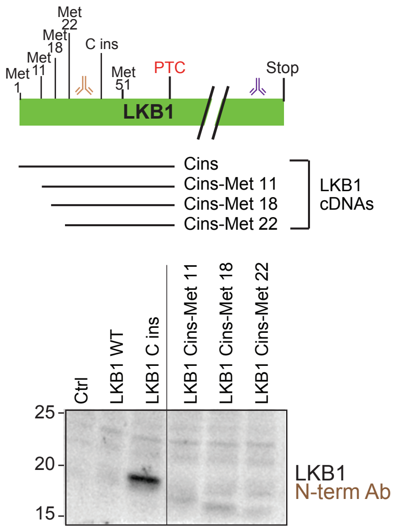

Supplemental figure 3. Leaky scanning does not contribute to the CRISPR/Cas9-mediated production of novel proteins. Overexpression constructs of *LKB1* gene with either WT sequence or C insertion initiating at methionine 1, 11, 18 or 22 were engineered and transiently transfected in Hela cells. Lysates generated from the cells were used to perform Western blot analysis with N-terminal LKB1 antibody. Source data are provided as a Source Data file.

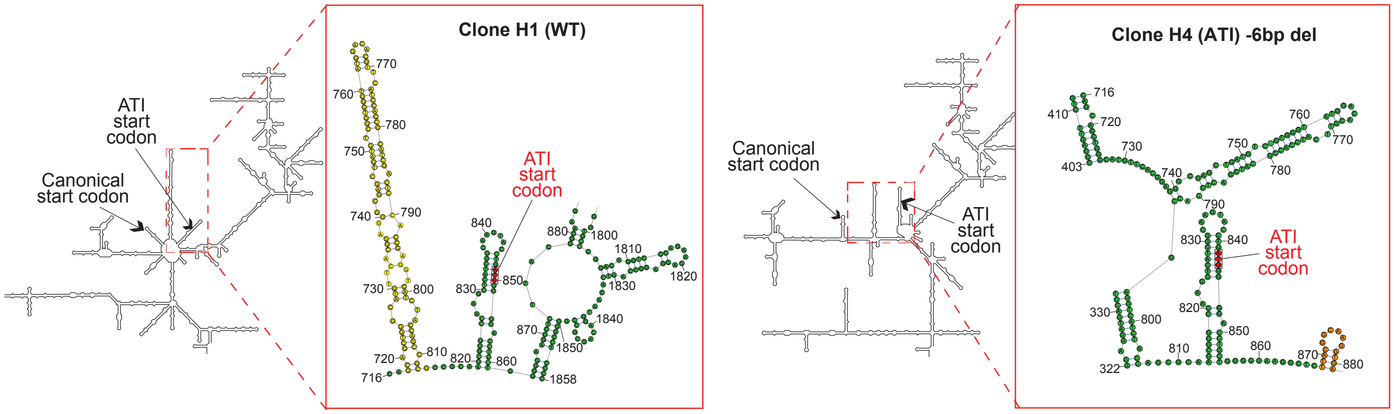

Supplemental figure 4. RNA secondary structure rearrangement triggered by CRISPR/Cas9-generated INDELs likely results in ATI. We used TurboFold II[1], an algorithm that predicts RNA secondary structures conserved in a set of homologous RNA sequences and aligns the sequences, to model the possible RNA structures of several mutant clones that generated the LKB1 Met51 initiated protein compared to those that did not. RNA structures of clone H1 (WT) and clone H4 (6 bp del) are representative of the clones without the ATI LKB1 protein or with the ATI LKB1 protein, respectively. The nucleotides in yellow marks the 5' hairpin structure in WT clone H1 and the nucleotides in orange marks the 3' hairpin structure in ATI LKB1 clone H4. Structures in the 5' untranslated region (UTR) near the 5'm7G cap have been shown to inhibit translation initiation[2, 3, 4] although very stable hairpins also can inhibit translation initiation farther away from the 5'm7G cap3. Conversely, hairpin structures 3' to AUG start codons can facilitate translation initiation, even at non-canonical start sites[5, 6, 7].

Clones without LKB1-45 KD protein

Methionine 51

```
> Clone H1 (WT)
GCAGCAGCTGGGCATGTTCACGGAGGGCGAGCTGATGTCGGTGGGTATGGACACGTTCATCCACCGCATCGACTCCACCGAGGTCATCTACCAGCCGCGC--CGCAAGCGGGCCAAGCTCATCGGCAAGTACCTGATGGGGGACCTGCTGGGGGAAGGCTCTTACGGCAAGGTGAA
...((.(((((..(((..(.(((..(..(((.(((((.(((((((((((....)))..))))))))))))).)))).))).)..)))...))))).))........((((((((...((((((((.......))))))))).)))))).((((.((((((....((((..(((((
> Clone H6 (9bp del, 3 bp ins)
GCAGCAGCTGGGCATGTTCACGGAGGGCGAGCTGATGTCGGTGGGTATGGACACGTTCATCCACCGCATCGACTCCACCGAGGTCATCTACCAGCCGCG----GCT----GGCCAAGCTCATCGGCAAGTACCTGATGGGGGACCTGCTGGGGGAAGGCTCTTACGGCAAGGTGAA
...((.(((((..(((..(.(((..(..(((.(((((.(((((((((((....)))..))))))))))))).)))).))).)..)))...))))).)).....((.....((((...((((((((.......))))))))).)).))..((((.((((((....((((..(((((
> Clone H8 (3 bp del)
GCAGCAGCTGGGCATGTTCACGGAGGGCGAGCTGATGTCGGTGGGTATGGACACGTTCATCCACCGCATCGACTCCACCGAGGTCATCTACCAGCCGC-----GCAAGCGGGCCAAGCTCATCGGCAAGTACCTGATGGGGGACCTGCTGGGGGAAGGCTCTTACGGCAAGGTGAA
...((.(((((..(((..(.(((..(..(((.(((((.(((((((((((....)))..))))))))))))).)))).))).)..)))...))))).))........((((((((...((((((((.......))))))))).)))))).((((.((((((....((((..(((((
> Clone H7 (39 bp ins)
GCAGCAGCTGGGCATGTTCACGGAGGGCGAGCTGATGTCGGTGGGTATGGACACGTTCATCCACCGCATCGACTCCACCGAGGTCATCTACCAGCCGCGC--TGGGACCCAAAAAAATATATCGGCAAGTACCTGATGGGGGACCTGCTGGGGGAAGGCTCTTACGGCAAGGTGAA
.......((((.....)).))).))))))).((((.(((((((((((....)))..))))))))))))..(((....)))........((((.((((..((((.(((.........((((((.......)))))).))).)).....((((.((((((....((((..(((((
```

Clones with LKB1-45 KD protein

```
> Clone H2 (11 bp del)
GCAGCAGCTGGGCATGTTCACGGAGGGCGAGCTGATGTCGGTGGGTATGGACACGTTCATCCACCGCATCGACTCCACCGAGGTCATCTACCAGCCGC-------------GCCAAGCTCATCGGCAAGTACCTGATGGGGGACCTGCTGGGGGAAGGCTCTTACGGCAAGGTGAA
.))).)))))))............((..(((.(((((.(((((((((((....)))..)))))))))))))).)))..)).))))))))).)).(((................((...((((((((.......))))))))..........))...)))..((((......)))).
> Clone H3 (6 bp del)
GCAGCAGCTGGGCATGTTCACGGAGGGCGAGCTGATGTCGGTGGGTATGGACACGTTCATCCACCGCATCGACTCCACCGAGGTCATCTACCAGCCGCGC--------CGGGCCAAGCTCATCGGCAAGTACCTGATGGGGGACCTGCTGGGGGAAGGCTCTTACGGCAAGGTGAA
.))).)))))))............((..(((.(((((.(((((((((((....)))..)))))))))))))).)))..)).))))))))).)).(((...........((((((...((((((((.......))))))))).))))........)))..((((......)))).
> Clone H4 (6 bp del)
GCAGCAGCTGGGCATGTTCACGGAGGGCGAGCTGATGTCGGTGGGTATGGACACGTTCATCCACCGCATCGACTCCACCGAGGTCATCTACCAG--------CGCAAGCGGGCCAAGCTCATCGGCAAGTACCTGATGGGGGACCTGCTGGGGGAAGGCTCTTACGGCAAGGTGAA
.))).)))))))............((..(((.(((((.(((((((((((....)))..)))))))))))))).)))..)).))))))))).)).............((((((((...((((((((.......))))))))).))))))...........((((......)))).
> Clone M2.1 (1 bp ins)
GCAGCAGCTGGGCATGTTCACGGAGGGCGAGCTGATGTCGGTGGGTATGGACACGTTCATCCACCGCATCGACTCCACCGAGGTCATCTACCAGCCGCGCC---- AAGCGGGCCAAGCTCATCGGCAAGTACCTGATGGGGGACCTGCTGGGGGAAGGCTCTTACGGCAAGGTGAA
.))).)))))))............((..(((.(((((.(((((((((((....)))..)))))))))))))).)))..)).))))))))).)).(((...((.....((((((((...((((((((.......))))))))).)))))))..))...)))..((((......)))).
> Clone M2.2 (2 bp del)
GCAGCAGCTGGGCATGTTCACGGAGGGCGAGCTGATGTCGGTGGGTATGGACACGTTCATCCACCGCATCGACTCCACCGAGGTCATCTACCAGCCGCGCC- CGCAAGCGGGCCAAGCTCATCGGCAAGTACCTGATGGGGGACCTGCTGGGGGAAGGCTCTTACGGCAAGGTGAA
.))).)))))))............((..(((.(((((.(((((((((((....)))..)))))))))))))).)))..)).))))))))).)).(((...((.(.(.((((((((...((((((((.......))))))))).))))))).)))..)))..((((......)))).
> Clone M3.1 (2 bp ins)
GCAGCAGCTGGGCATGTTCACGGAGGGCGAGCTGATGTCGGTGGGTATGGACACGTTCATCCACCGCATCGACTCCACCGAGGTCATCTACC AGCCGCGCCGCGCAAGCGGGCCAAGCTCATCGGCAAGTACCTGATGGGGGACCTGCTGGGGGAAGGCTCTTACGGCAAGGTGAA
.))).)))))))............((..(((.(((((.(((((((((((....)))..)))))))))))))).)))..)).))))))))).)).(((...((.(...((((((((...((((((((.......))))))))).))))))).))...)))..((((......)))).
> Clone M4.1 (15 bp del)
GCAGCAGCTGGGCATGTTCACGGAGGGCGAGCTGATGTCGGTGGGTATGGACACGTTCATCCACCGCATCGACTCCACCGAGGTC-----------------CGCAAGCGGGCCAAGCTCATCGGCAAGTACCTGATGGGGGACCTGCTGGGGGAAGGCTCTTACGGCAAGGTGAA
.))).)))))))............((..(((.(((((.(((((((((((....)))..)))))))))))))).)))..)).....(................(.(.((((((((...((((((((.......))))))))).))))))).)).........((((......)))).
> Clone H5 (18 bp del)
GCAGCAGCTGGGCATGTTCACGGAGGGCGAGCTGATGTCGGTGGGTATGGACACGTTCATCCACCGCATCGACTCCACCGAG--------------------CGCAAGCGGGCCAAGCTCATCGGCAAGTACCTGATGGGGGACCTGCTGGGGGAAGGCTCTTACGGCAAGGTGAA
.))).))))))))))).))).))).((..(((.(((((.(((((((((((....)))..)))))))))))))).)))..)).........................((((((((...((((((((.......))))))))).))))))...........((((......)))).
```

Supplemental figure 5. RNA secondary structure analysis predicts motifs and features unique to CRISPR/Cas9-edited clones with ATI. Turbofold II, an RNA structure prediction algorithm, was used to predict secondary structures for 12 different mutations in LKB1 generated by CRISPR/Cas9 gene editing using an sgRNA targeting exon 1 in MIA and HAP1 cells. Mutations in clones H1, H6, H7 and H8 were not associated with ATI. Mutations in clones H2, H3, H4, H5, M2.1, M2.2, M3.1, and M4.1 were associated with ATI. The predicted RNA structures are shown by dots and brackets along with alignments, where dot represents an unpaired nucleotide and bracket represents paired nucleotides. The structures of non-ATI clones H1, H6 and H8 show a unique helix (in yellow background) located before the Methionine 51 site (ATG highlighted in red). All eight sequences associated with ATI are predicted to form a conserved hairpin loop 3' to Methionine 51 start site (TTACGGCAAGGTGA, in orange).

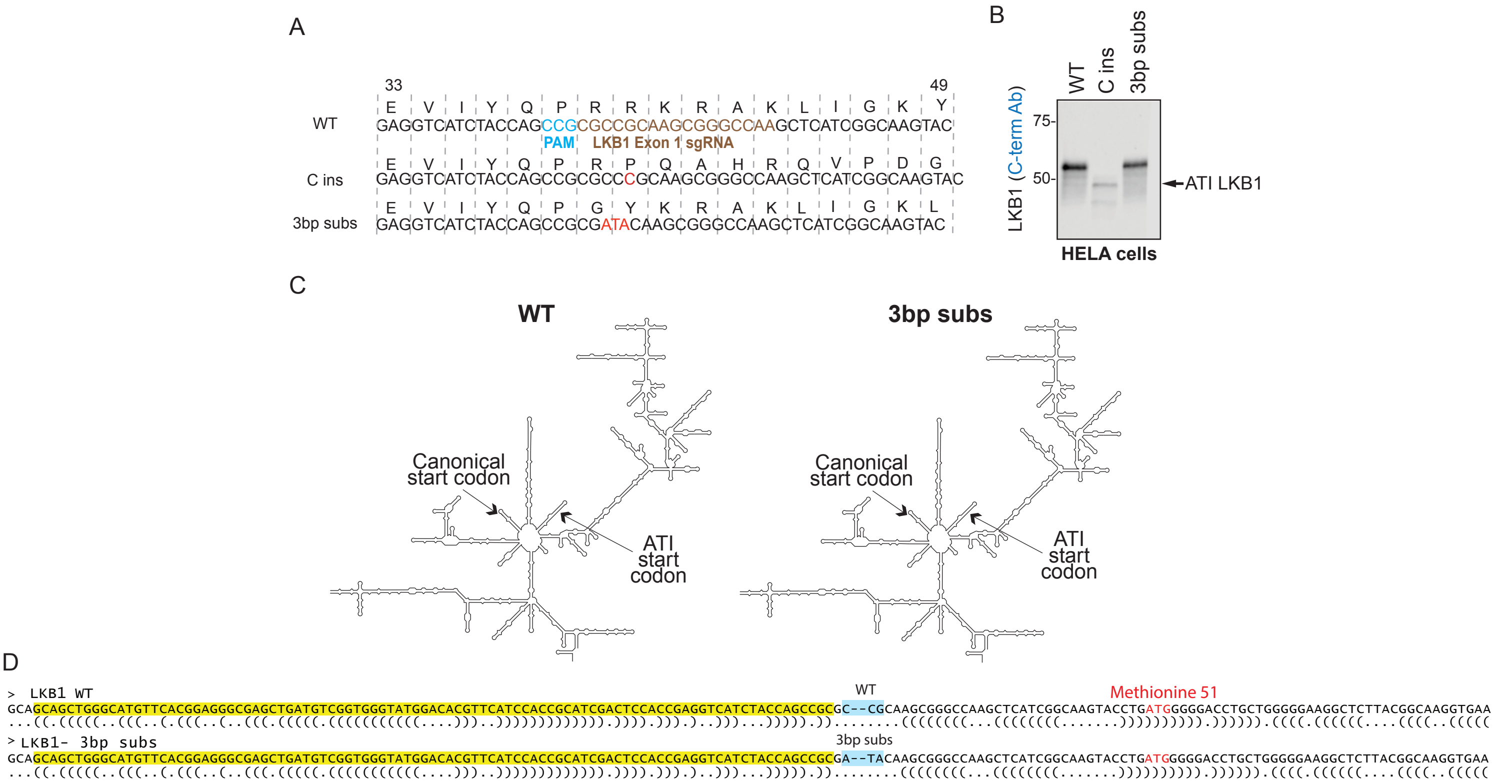

Supplemental figure 6. Point mutagenesis does not replicate the ATI-inducing effect of INDELs introduced by CRISPR/Cas9. (A) Expression plasmids with WT LKB1, the C insertion introduced by CRISPR/Cas9 in edited cell lines associated with ATI of LKB1, or a 3 bp substitution were engineered. **(B)** Lysates from HELA cells transiently transfected with LKB1 plasmids described in “A” were used to perform Western blot analysis with C-terminal LKB1 antibody. **(C)** No change in predicted RNA secondary structure was observed with the 3 bp substitution. **(D)** The predicted RNA structure represented by dot and brackets for WT LKB1 and LKB1 with the 3 bp substitution.

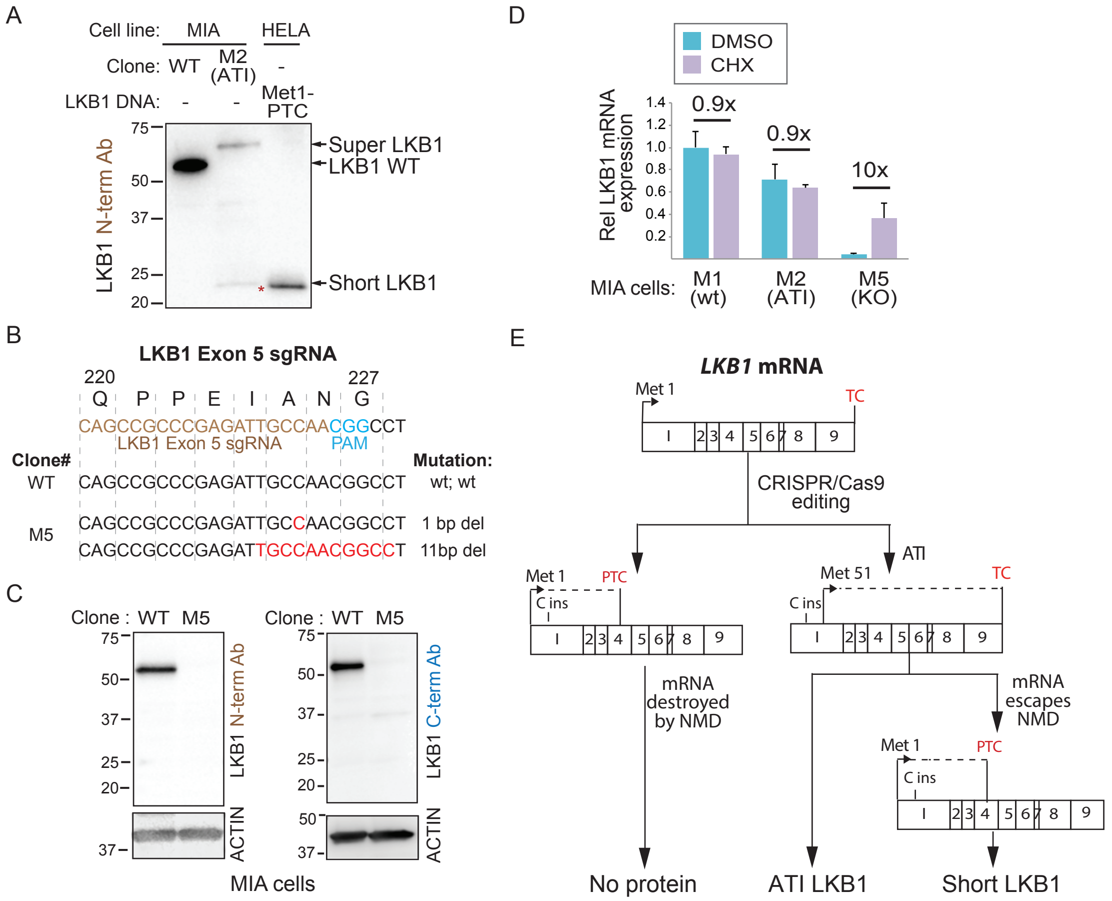

Supplemental figure 7. ATI bypasses the induction of NMD in the presence of a pre-mature termination codon (PTC). (A) CRISPR/-Cas9-edited MIA clones targeting LKB1 exhibiting ATI express a C-terminally truncated LKB1 protein (Short LKB1). Lysates from MIA LKB1 clones were subjected to Western blot analysis using an LKB1 antibody recognizing an N-terminus localized epitope. An LKB1 overexpression construct with canonical start site and the predicted PTC introduced by CRISPR/Cas9-editing was transiently transfected in HELA cells and analyzed along with the MIA clones. **(B)** Targeting an internal LKB1 exon as a strategy for generating MIA LKB1 KO cells. Genomic sequence of a MIA clone M5 edited with LKB1 exon 5 sgRNA indicates presence of a frameshift alteration. **(C)** Engineering MIA cells with no detectable expression of LKB1 proteins. Western blot analysis of MIA clone M5 was performed using LKB1 N- and C-terminal antibodies. **(D)** ATI prevents nonsense-medicated decay (NMD). MIA WT, clone M2 (ATI) and clone M5 (LKB1 KO) cells were treated with DMSO or 10 μg/ml CHX for 6 hrs and isolated cDNAs was subjected to quantitative RT-PCR (qPCR) using primers flanking Exons 3 and 4. The MIA clone with a non-ATI inducing mutation in LKB1 exhibits a 10-fold change in mRNA abundance upon CHX treatment. CHX treatment has no effect on LKB1 mRNA abundance in cells with ATI. **(E)** Production of novel LKB1 proteins as a consequence of a CRISPR/Cas9-induced INDEL. Source data are provided as a Source Data file.

Genes	Targeted exon	CRISPR-introduced INDELs	INDELs hit ESEs?	Exon skipped?
CTNNB1	3	4bps del	No	No
AXIN1	2	1 bps ins	No	No
LRP6	2	5 bps del	No	No
TBK1	1	2 bps ins	No	No
BAP1	5	109 bps ins	No	No
TLE3	7	2 bps del	No	Yes
PPM1A	1	5 bps del	No	No
BCL2L2	1	5 bps del	No	No
SUFU	3	11bps del, 1bp ins	No	No
SUFU	8	1 bps del	No	No
RICTOR	5	10 bps del	Yes	No
VPS35	5	11 bps del	Yes	No
TOP1	6	8 bps del	Yes	Yes
SIRT1	4	20 bps del	Yes	Yes
PTEN	1	5 bps del	Yes	No
SUFU	2	5 bps del, 11 bp del	Yes	Yes
SUFU	3	26 bps del, 2 bps del	Yes	Yes
SUFU	8	28 bps del, 1 bp ins	Yes	Yes
SUFU	8	11 bp del	Yes	Yes
SUFU	8	2 bps del	Yes	Yes
SUFU	8	1 bp del	Yes	Yes
SUFU	8	46 bps ins	Yes	Yes
SUFU	8	61 bps ins	Yes	Yes
SUFU	8	1 bp ins	Yes	Yes

% Exon skipped when ESE is compromised: 78% (11/14)

% Exon skipped when ESE is not compromised: 10% (1/10)

Supplemental figure 8 CRISPR/Cas9-edited cells with different mutations analyzed for ESE disruption and exon skipping. List of 24 CRISPR/Cas9-edited cells from Horizon Discovery and *de novo* engineering with indicated targeted exons, frameshift inducing mutations, predicted impact on ESEs, and exon skipping status derived from RT-PCR analysis.

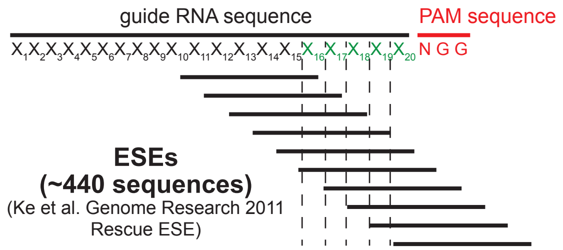

Supplemental figure 9. ESE sequence evaluation using the CRISPinatoR. The CRISPinatoR algorithm selects for sgRNAs containing ESEs within 5bp 5' to the PAM sequence. 440 putative ESE sequences previously described (http://genes.mit.edu/burgelab/rescue-ese/ and Ke et al.) were used to develop the CRISPinatoR algorithm.

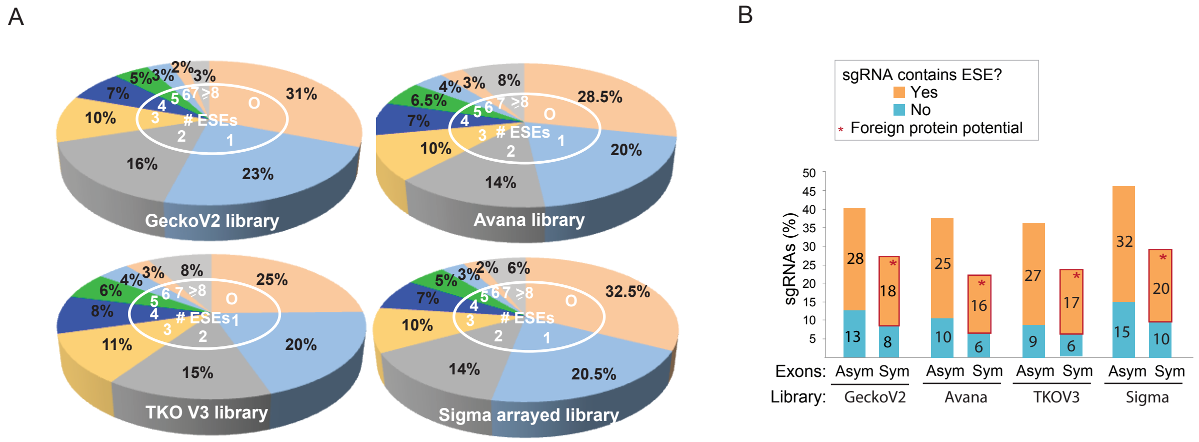

Supplemental figure 10. ESE sequence targeting prevalence in genome-wide CRISPR/Cas9 libraries. (A) ESE annotation in commercial CRISPR libraries. Putative ESEs embedded within the sgRNA sequences present in 4 different CRISPR libraries were analyzed: Gecko V2, Avana, TKO V3, and Sanger. **(B)** Genome-wide CRISPR/Cas9 screening libraries do not avoid inadvertent targeting of ESEs. Exon symmetry and ESE targeting potential were calculated for the different CRISPR/Cas9 libraries. sgRNAs with putative ESEs targeting symmetric exons (red box) have the potential to generate foreign proteins.

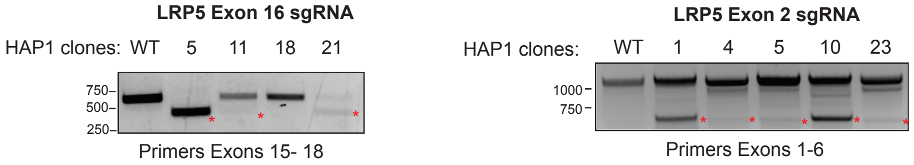

Supplemental figure 11. Induction of exon skipping with targeted disruption of ESEs in an asymmetric or symmetric exon of LRP5. (A) RT-PCR analysis of HAP1 clones edited with the LRP5 Exon 16 sgRNA. **(B)** RT-PCR analysis of HAP1 clones edited with the LRP5 Exon 2 sgRNA. Source data are provided as a Source Data file.

Supplemental Table 1. Catalog numbers for Horizon Discovery's HAP1 cell lines with INDELs

Gene KO	Catalog number
AXIN1	HZGHC000355c011
BAP1	HZGHC003319c004
BCL2L2	HZGHC003084c007
CTNNB1	HZGHC001032c004
LRP6	HZGHC000598c007
PPM1A	HZGHC003112c011
PTEN	HZGHC000624c009
RICTOR	HZGHC001020c010
SIRT1	HZGHC001008c008
TBK1	HZGHC000031c012
TLE3	HZGHC004203c002
TOP1	HZGHC001210c003
VPS35	HZGHC000863c012

Supplemental Table 2. Antibodies used for Western blot analysis of CRISPR-edited cell lines.

Genes	Antibody #1	Antibody #2
AXIN1	Cell Signaling 2087	Cell Signaling 2074
BAP1	Cell signaling 13271	Bethyl A302-243A-T
BCL2L2	Cell signaling 2724	LS Bioscience LS-C382259-100
CTNNB1	BD Biosciences 610153	Sigma-Aldrich C2206
LRP6	Cell Signaling 2560	Santa cruz sc-25317
PPM1A	Abcam 14824	LS Bioscience LS-C169090-100
PTEN	Invitrogen 44-1064	Cell signaling 9552
RICTOR	Bethyl A300-459A	Cell Signaling 9476
SIRT1	Bethyl A300-688A	Cell Signaling (D739) 2493
TBK1	Abcam ab40676	Cell signaling 3504
TLE3	Cell signaling 4681	Santa Cruz (M-201) sc-9124
TOP1	Bethyl A302-589A	Bethyl 302-590A
VPS35	Bethyl A304-727-A	Genetex GTX108058
LKB1	Santa Cruz (E-9) s-374334	Cell signaling 3050
SUFU	Abcam 52913	Cell signaling 2520
LRP5	Cell Signaling (D80F2) 5731	Santa Cruz (B-9) sc-390267

Supplemental Table 3. Primers used for RT-PCR of CRISPR Cas9-edited cell lines.

Genes	Forward Primer		Reverse Primer	
	Primer Location	Primer Sequence	Primer Location	Primer Sequence
AXIN1	Exon 1	GGTATGAGCCTGAGGGC	Exon 3	GGAATGTGAGGTAGGGGC
BAP1	Exon 4	GGATGATACGTCCGTGATTGATGATG	Exon 9	CCTCTAGTACTGTCTGACGGTTCACC
BCL2L2	5' UTR	CAGCTCCTGCACCAGGAAAC	Exon 2	CCGTATAGAGCTGTGAACT
CTNNB1	5' UTR	GAAAATCCAGCGTGGACAAT	Exon 4	CATCTGAGGAGAACGCATGA
LRP6	Exon 1	GAGAAGAGAACGCGAGAAGG	Exon 4	TGTGCTCCAGTCAGTCCAGT
PPM1A	5' UTR	TCGGCCGACCAGGGACCT	Exon 2	TCACACTCATGTTGTCTCGAC
PTEN	5' UTR	GCAGCAGCCATTACCCG	Exon 2	CCTTCAAGTCTTTCTGCAGGAAATCC
RICTOR	Exon 2	GAACGTCCCGCTGGATCTGAC	Exon 9	GCTGTATCTGGACTATGTCTG
SIRT1	Exon 2	TCCTGTGAAAGTGATGAGGAG	Exon 7	ATGATAGCAAGCGGTTCATCA
TBK1	5'UTR	GGAAGTGTCCTGAGTCTCGAGG	Exon 3	CGCAAAACAATTAAGAATTCAGATTCTGGTAGTCC
TLE3	Exon 5	CAGATCATGCCTTTCCTGTCACAAG	Exon 11	CCAAGGTCTTTGGTCTTGGAGGAAG
TOP1	Exon 3	AGATCGAGAACACCGGCACAA	Exon 9	CCACTTGATGCCTTCAGGATA
VPS35	Exon 1	GTGAAGGTCCAGTCATTCCAA	Exon 7	CCTACAGTTTACAACTTGCTC
LKB1	5' UTR	AGGGCTGGCGGCGGGACTCCAG	Exon 4	TTTGAGGGTGCCACCGGTGGTG
LKB1	Cryptic exon Fwd	CAACGGTACCAGCATGGAGGTGGTGGA	Cryptic exon Rev	TGGTCTCGAGAGAGTGGCCAGGAGTTCTTCA
LKB1	Exon 3	GTATATGGTGATGGAGTAC	Exon 4	TTTGAGGGTGCCACCGGTGGTG
SUFU	Exon 6	CTGACATGCGGAGGGGAGAGAC	Exon 10	GTGATGGCCATGTCACCTGTGATAC
SUFU	Exon 1	CCGCTCCAGGTTACCGCTATCG	Exon 5	CTGCTGGGCTGAGTGTAGCTC
SUFU	5' UTR	CATCGCCTCGGGGAGTCTCACC	Exon 4	TACCCCAAAGGGTGTCTGCACG
LRP5	Exon 15	CTGTACTTCACCAACATGCA	Exon 18	CTCCTCGTCGCTCTGGTCAT
LRP5	5' UTR	ATGGAGCCCGAGTGAGCG	Exon 6	CAGCACGATGTCGGTGAAGT

Supplemental References

1. Tan Z, Fu Y, Sharma G, Mathews DH. TurboFold II: RNA structural alignment and secondary structure prediction informed by multiple homologs. Nucleic Acids Res 45, 11570-11581 (2017).
2. Jenkins RH, Bennagi R, Martin J, Phillips AO, Redman JE, Fraser DJ. A conserved stem loop motif in the 5'untranslated region regulates transforming growth factor-beta(1) translation. PLoS ONE 5, e12283 (2010).
3. Kozak M. Circumstances and mechanisms of inhibition of translation by secondary structure in eucaryotic mRNAs. Mol Cell Biol 9, 5134-5142 (1989).
4. Svitkin YV, et al. The requirement for eukaryotic initiation factor 4A (eIF4A) in translation is in direct proportion to the degree of mRNA 5' secondary structure. RNA 7, 382-394 (2001).
5. Jagodnik J, Chiaruttini C, Guillier M. Stem-Loop Structures within mRNA Coding Sequences Activate Translation Initiation and Mediate Control by Small Regulatory RNAs. Mol Cell 68, 158-170 e153 (2017).
6. Kozak M. Downstream secondary structure facilitates recognition of initiator codons by eukaryotic ribosomes. Proc Natl Acad Sci U S A 87, 8301-8305 (1990).
7. Paulus M, Haslbeck M, Watzele M. RNA stem-loop enhanced expression of previously non-expressible genes. Nucleic Acids Res 32, e78 (2004).
