# Supplementary material for: CRISPR-Cas9-based mutagenesis frequently provokes on-target mRNA misregulation
Source: Nat Commun. 2019 Sep 6;10:4056. doi: 10.1038/s41467-019-12028-5 (PMC6731291; doi:10.1038/s41467-019-12028-5)
Supplement: Supplementary file 3 — Reporting Summary [file 41467_2019_12028_MOESM3_ESM.pdf]

nature research

Corresponding author(s): Lawrence Lum and Tae Hyun Hwang

Last updated by author(s): May 11, 2019

Reporting Summary

Nature Research wishes to improve the reproducibility of the work that we publish. This form provides structure for consistency and transparency in reporting. For further information on Nature Research policies, see Authors & Referees and the Editorial Policy Checklist.

Statistics

For all statistical analyses, confirm that the following items are present in the figure legend, table legend, main text, or Methods section.

n/a	Confirmed	
☐	☒	The exact sample size (*n*) for each experimental group/condition, given as a discrete number and unit of measurement
☐	☒	A statement on whether measurements were taken from distinct samples or whether the same sample was measured repeatedly
☐	☒	The statistical test(s) used AND whether they are one- or two-sided<br>*Only common tests should be described solely by name; describe more complex techniques in the Methods section.*
☒	☐	A description of all covariates tested
☒	☐	A description of any assumptions or corrections, such as tests of normality and adjustment for multiple comparisons
☒	☐	A full description of the statistical parameters including central tendency (e.g. means) or other basic estimates (e.g. regression coefficient) AND variation (e.g. standard deviation) or associated estimates of uncertainty (e.g. confidence intervals)
☒	☐	For null hypothesis testing, the test statistic (e.g. *F*, *t*, *r*) with confidence intervals, effect sizes, degrees of freedom and *P* value noted<br>*Give P values as exact values whenever suitable.*
☒	☐	For Bayesian analysis, information on the choice of priors and Markov chain Monte Carlo settings
☒	☐	For hierarchical and complex designs, identification of the appropriate level for tests and full reporting of outcomes
☒	☐	Estimates of effect sizes (e.g. Cohen's *d*, Pearson's *r*), indicating how they were calculated

Our web collection on statistics for biologists contains articles on many of the points above.

Software and code

Policy information about availability of computer code

Data collection	No software was used to collect data.
Data analysis	TurboFoldII was used to generate anticipated secondary structures for RNA - description of this software is published (Nucleic Acids Res. 2017 Nov 16;45(20):11570-11581. doi: 10.1093/nar/gkx815.) and can also be found online (http://rna.urmc.rochester.edu/ RNAstructure.html).<br>Description of the CRISPinator software for identifying exon splicing enhancers (ESEs) and sgRNAs for Cas9 that target these ESEs can be found on line (http://www.crispinator.com/about/) and are described in the methods section. In addition, a manuscript describing the CRispinator algorithm in more detail has been submitted.

For manuscripts utilizing custom algorithms or software that are central to the research but not yet described in published literature, software must be made available to editors/reviewers. We strongly encourage code deposition in a community repository (e.g. GitHub). See the Nature Research guidelines for submitting code & software for further information.

Data

Policy information about availability of data

All manuscripts must include a data availability statement. This statement should provide the following information, where applicable:

- Accession codes, unique identifiers, or web links for publicly available datasets
- A list of figures that have associated raw data
- A description of any restrictions on data availability

The data that support the findings of this study are available from the corresponding author upon reasonable request.

Field-specific reporting

Please select the one below that is the best fit for your research. If you are not sure, read the appropriate sections before making your selection.

☒ Life sciences ☐ Behavioural & social sciences ☐ Ecological, evolutionary & environmental sciences

For a reference copy of the document with all sections, see nature.com/documents/nr-reporting-summary-flat.pdf

Life sciences study design

All studies must disclose on these points even when the disclosure is negative.

Sample size	No sample size calculations were made for the bulk of the studies given it was a survey of "random" genes selected for knockout campaigns and cell lines from a mixture of commercial sources or internally engineered lines. For reporter studies (such as Fig. 5H), each condition was performed in triplicate which is a generally accepted sample size for such studies. Western blot results were confirmed minimally twice using different cell lysate samples. For RNA splicing analysis, novel amplicons observed were confirmed by sequencing (Supplemental Table 4) to account for their presence and deviation from cDNA sequence derived from wildtype/parental cells.
Data exclusions	Raw image files are included in a supplemental figure ("Raw images file". Some commercially available antibodies that were not able to detect their targeted protein were excluded from the study.
Replication	All experiments were conducted at least twice and attempts at replication were successful.
Randomization	Allocation was not random. Various researchers that were tasked to generate or acquire various engineered cell lines reported their initial findings on mRNA or protein expression misregulation. Subsequent MOA studies were not randomly allocated.
Blinding	Blinding was not relevant to the bulk of our study - the results reported were all attempts to evaluate commercially available cell lines or to engineer specific gene knockouts. For the computationally based predictions of RNA structure and impact of insertion-deletion mutations on exon splicing enhancers, our collaborators were blinded to experimental data for alternative translation initiation and mRNA splicing alterations.

Reporting for specific materials, systems and methods

We require information from authors about some types of materials, experimental systems and methods used in many studies. Here, indicate whether each material, system or method listed is relevant to your study. If you are not sure if a list item applies to your research, read the appropriate section before selecting a response.

Materials & experimental systems

n/a	Involved in the study	
☐	☒	Antibodies
☐	☒	Eukaryotic cell lines
☒	☐	Palaeontology
☒	☐	Animals and other organisms
☒	☐	Human research participants
☒	☐	Clinical data

Methods

n/a	Involved in the study	
☒	☐	ChIP-seq
☒	☐	Flow cytometry
☒	☐	MRI-based neuroimaging

Antibodies

Antibodies used	Many commercially available antibodies were used in this study - their source is described in detail in Supplemental Table 2.
Validation	Antibodies were all validated using CRISPR knockout cell lines throughout the manuscript and supplemental figures.

Eukaryotic cell lines

Policy information about cell lines

Cell line source(s)	Cell lines were purchased from ATCC or Horizon Discovery as described in the methods section and Supp. table 1.
Authentication	None of the cell lines were authenticated. All were purchased directly from commercial suppliers.
Mycoplasma contamination	All cell lines were confirmed to be mycoplasma free.
Commonly misidentified lines (See ICLAC register)	Hela cells are commonly misidentified and were used in these studies.
