# Supplementary material for: CRISPR-Cas9-based mutagenesis frequently provokes on-target mRNA misregulation
Source: Nat Commun. 2019 Sep 6;10:4056. doi: 10.1038/s41467-019-12028-5 (PMC6731291; doi:10.1038/s41467-019-12028-5)
Supplement: Supplementary file 4 — Description of Additional Supplementary Files [file 41467_2019_12028_MOESM4_ESM.pdf]

Description of Additional Supplementary Files

Supplementary Data 1
Description: Sequencing results for cDNA transcripts.
