# Supplementary material for: CRISPR-Cas9-based mutagenesis frequently provokes on-target mRNA misregulation
Source: Nat Commun. 2019 Sep 6;10:4056. doi: 10.1038/s41467-019-12028-5 (PMC6731291; doi:10.1038/s41467-019-12028-5)
Supplement: Supplementary file 6 — Source Data [file 41467_2019_12028_MOESM6_ESM.pdf]

CRISPR/Cas9-based mutagenesis frequently provokes on-target mRNA misregulation

Rubina Tuladhar, Yunku Yeu, John Tyler Piazza, Zhen Tan, Jean Rene Clemenceau, Xiaofeng Wu, Quinn Barrett, Jeremiah Herbert, David H. Mathews, James Kim, Tae Hyun Hwang, and Lawrence Lum.

Figure 1B

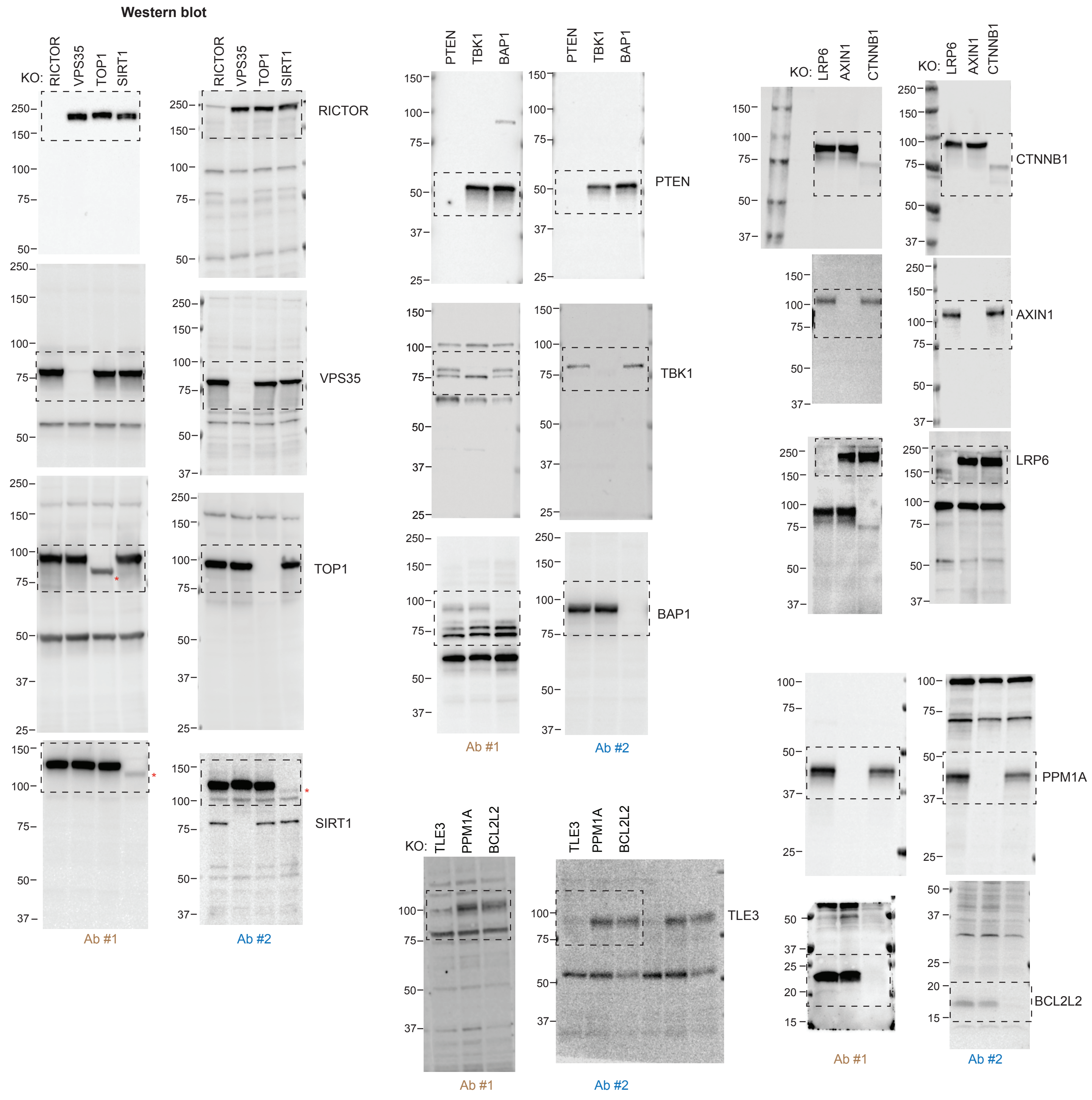
Western blot
KO: RICTOR VPS35 TOP1 SIRT1
RICTOR
VPS35
TOP1
SIRT1
Ab #1
Ab #2
PTEN TBK1 BAP1
PTEN
TBK1
BAP1
KO: TLE3 PPM1A BCL2L2
TLE3
KO: LRP6 AXIN1 CTNNB1
CTNNB1
AXIN1
LRP6
PPM1A
BCL2L2

Figure 1C

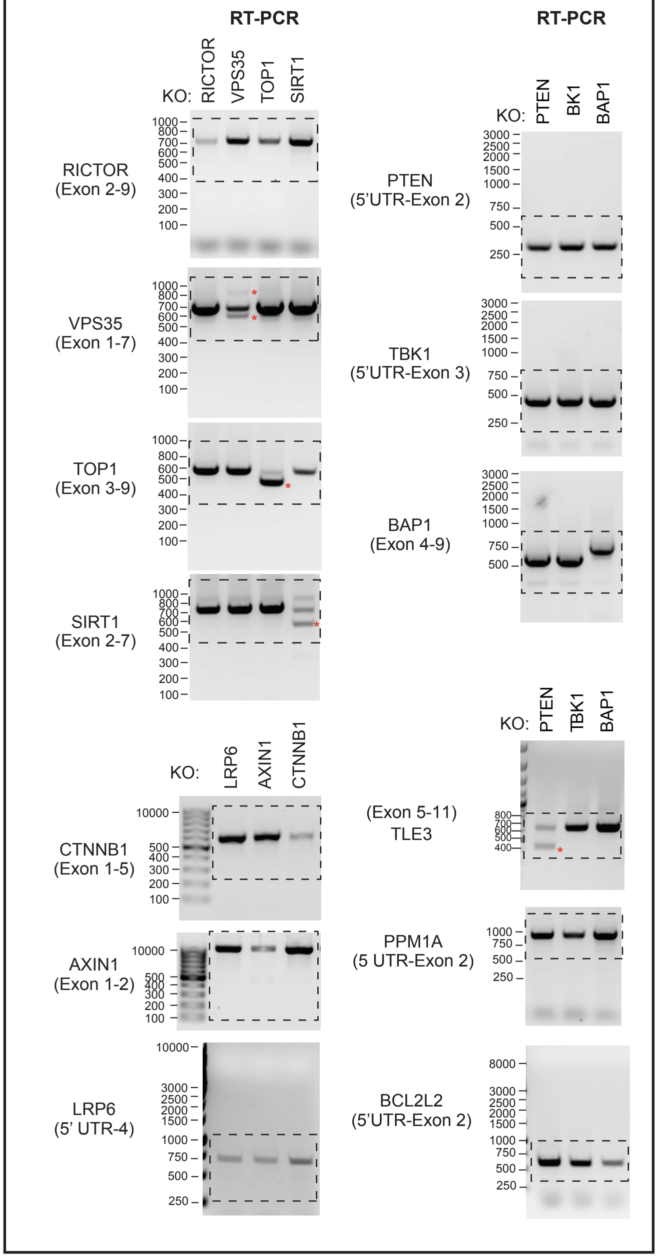

Figure 2A

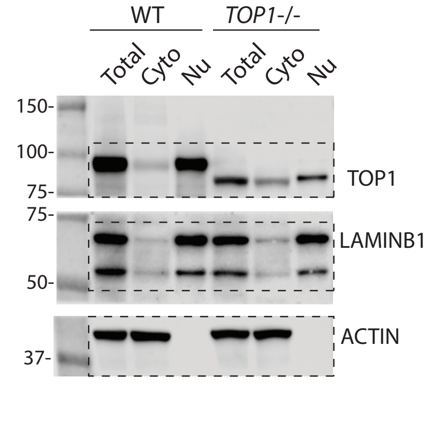

Figure 2B

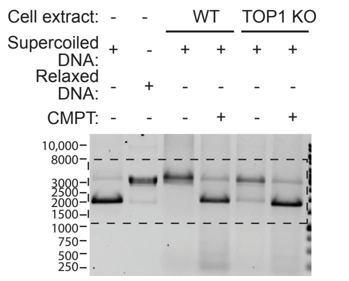

Figure 3B

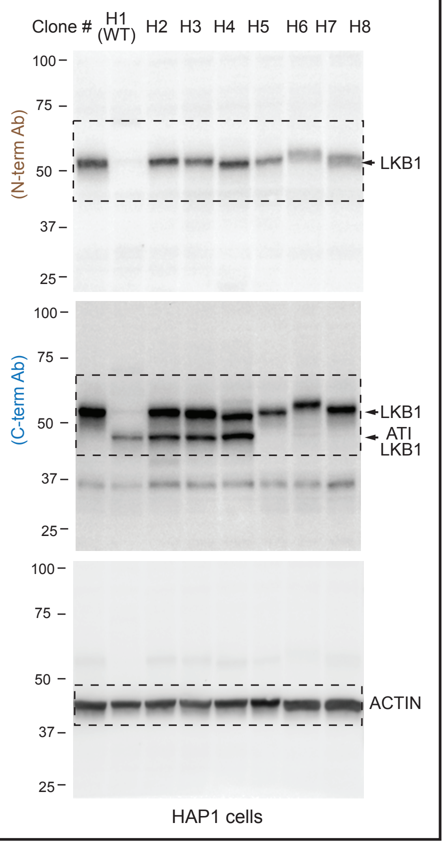

Figure 3C

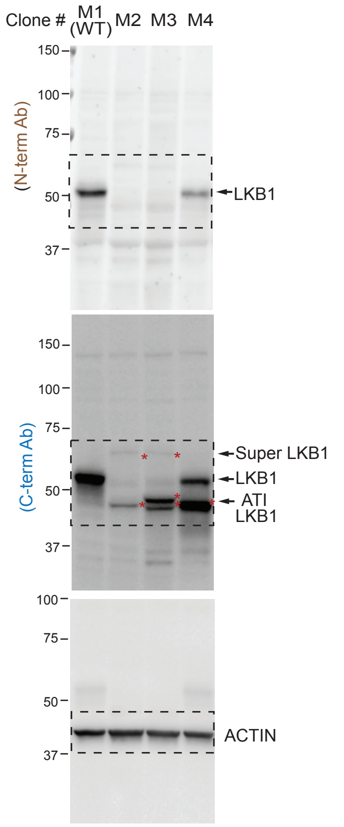

Figure 3E

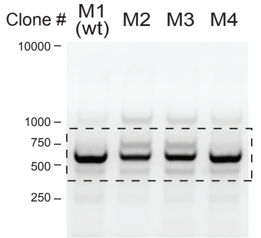

RT-PCR (5'UTR to Exon 4)
MIA Cells

Figure 3F

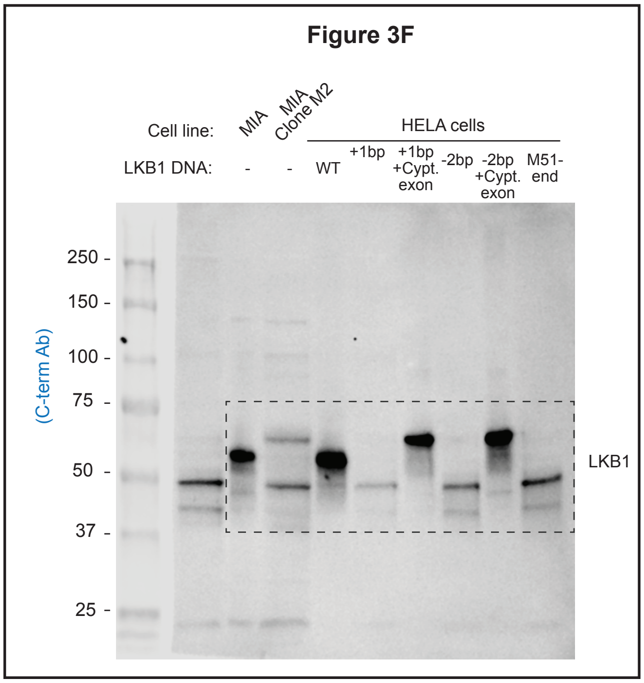

Figure 4B

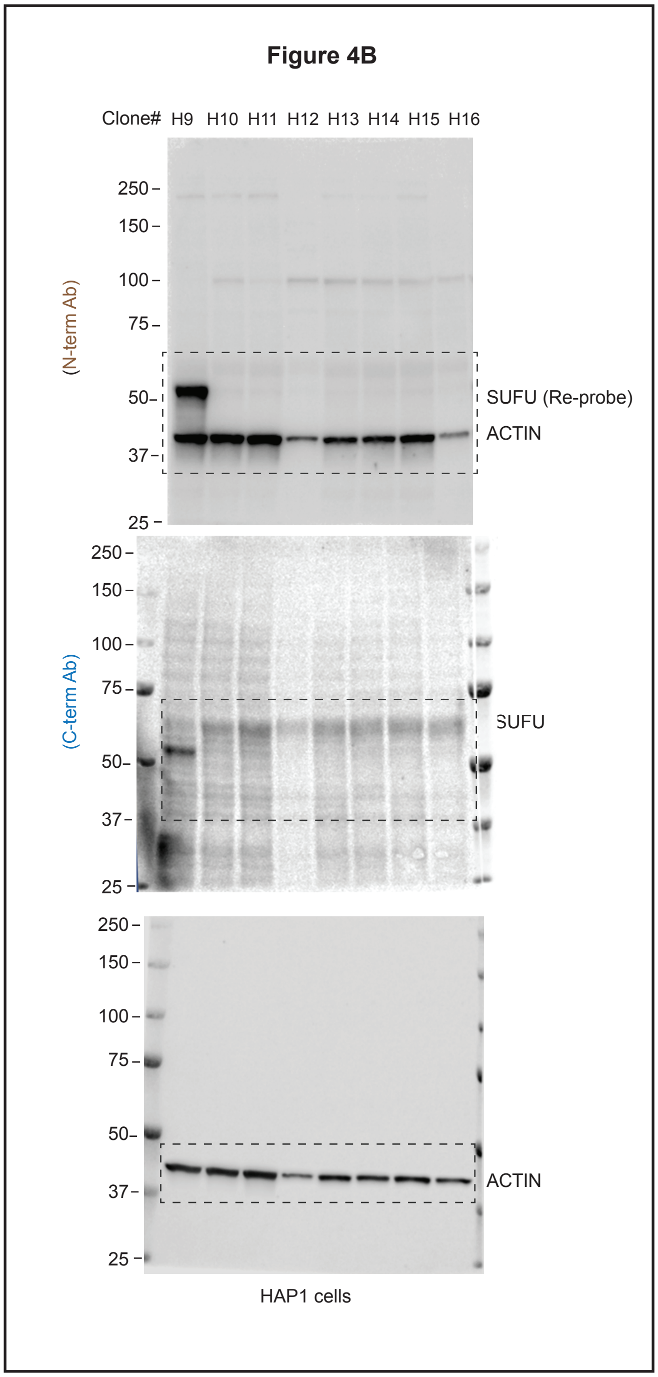

Figure 4C

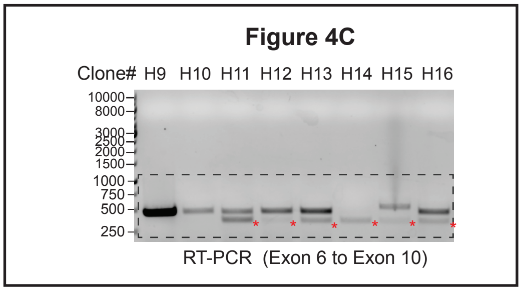

Figure 4F

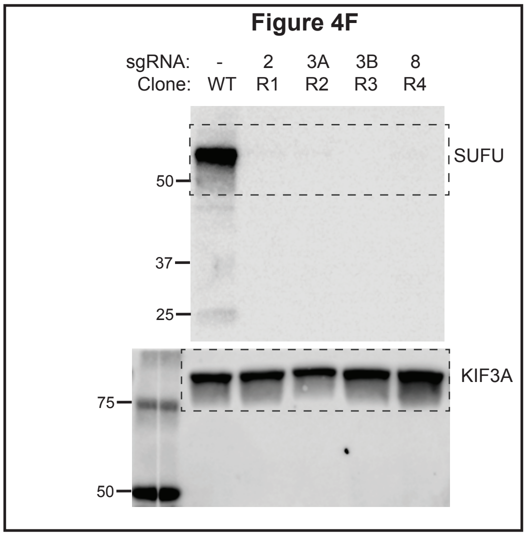

Figure 4H

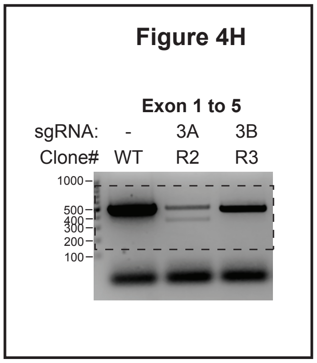

Figure 4J

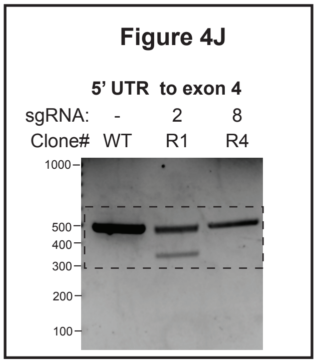

Figure 4L

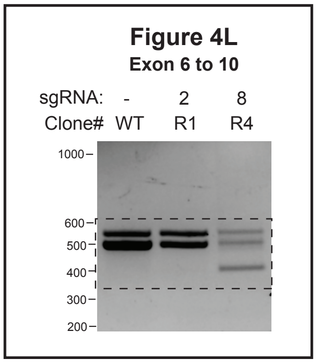

Figure 5D

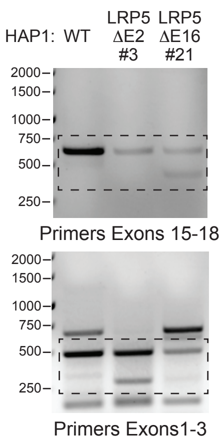

Figure 5E

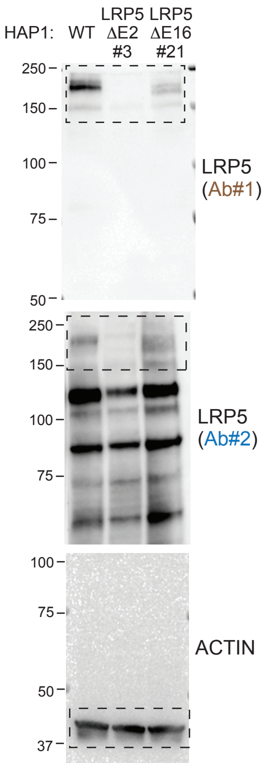

Figure 5F

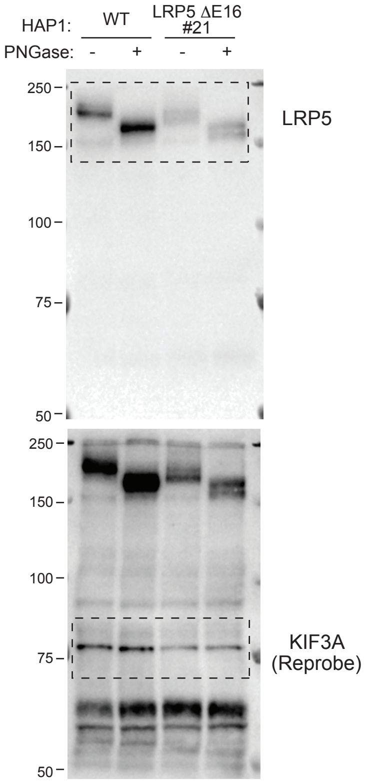

Supp. Fig 1A

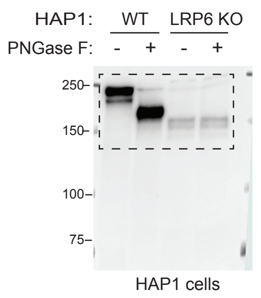

Supp. Fig 1B

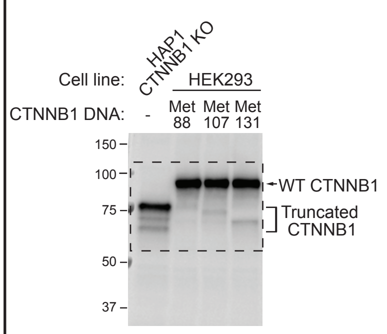

Supp. Fig 2A

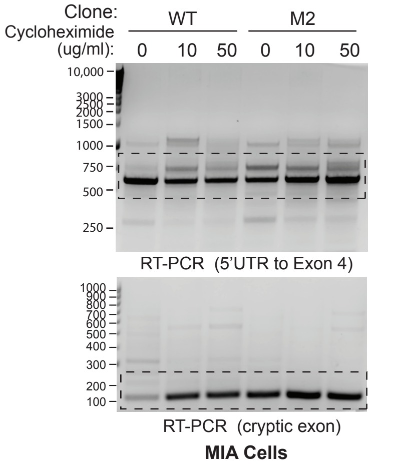

Supp. Fig 2B

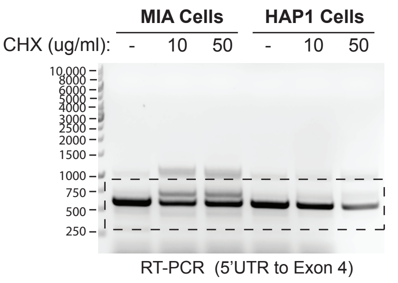

Supp. Fig 3

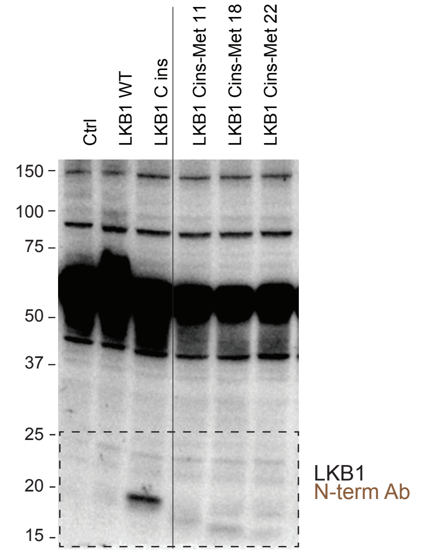

Supp. Fig. 7A

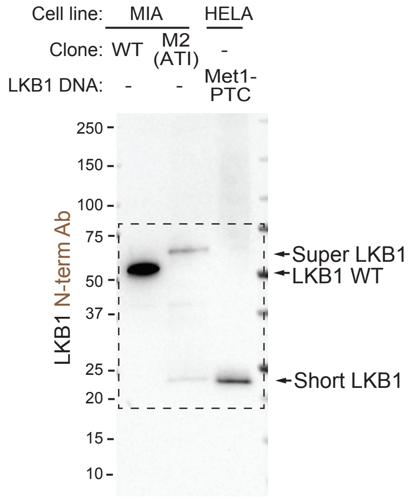

Supp. Fig. 7C

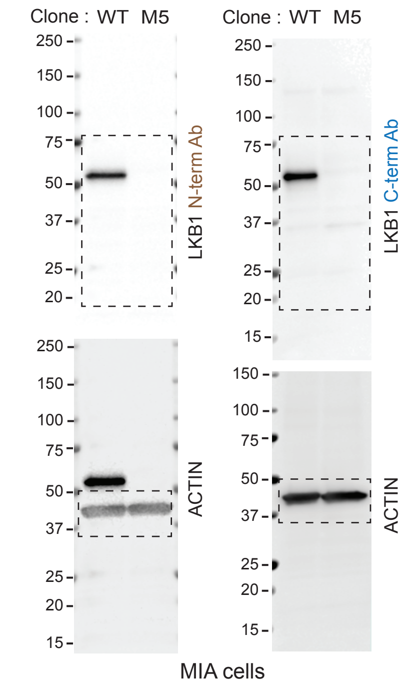

Supp. Fig. 11A

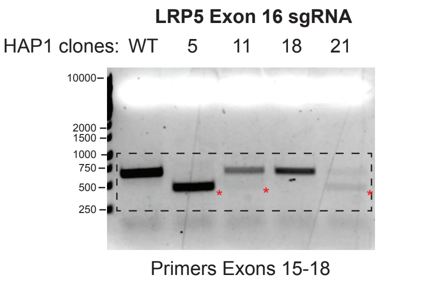

Supp. Fig.11B

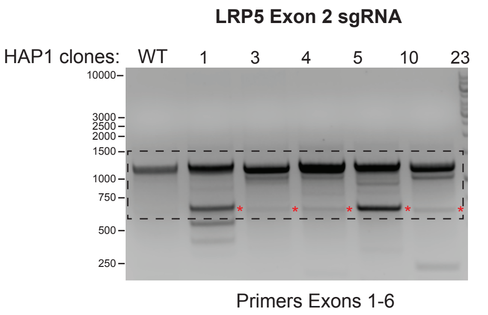

Raw data for bar graph in Figure 5H.

WT Cells			LRP5 ΔE2 #3			LRP5 ΔE16 #21		
DMSO	PORCNi	TNKSi	DMSO	PORCNi	TNKSi	DMSO	PORCNi	TNKSi
25269	22854	2488	3539	5208	864	17369	12000	2354
30984	20195	2725	2836	2583	876	13351	13770	2647
30401	21896	5106	1880	3350	649	14586	6806	2227
